# Patterns of HIV Self-Disclosure in the Oncology Setting

**DOI:** 10.1093/jncics/pkab058

**Published:** 2021-06-04

**Authors:** Lindsay N Fuzzell, Susan T Vadaparampil, Anna R Giuliano, Yifen Liu, Anna E Coghill

**Affiliations:** 1 Health Outcomes and Behavior, H. Lee Moffitt Cancer Center & Research Institute, Tampa, FL, USA; 2 Office of Community Outreach, Engagement, and Equity, H. Lee Moffitt Cancer Center & Research Institute, Tampa, FL, USA; 3 Center for Immunization and Infection Research in Cancer, H. Lee Moffitt Cancer Center & Research Institute, Tampa, FL, USA; 4 Cancer Epidemiology Program, H. Lee Moffitt Cancer Center & Research Institute, Tampa, FL, USA

## Abstract

Higher rates of cancer treatment toxicity and uniquely poor outcomes following a cancer diagnosis have been reported for persons living with HIV (PLWH). This highlights the importance of active HIV status ascertainment in the oncology setting. Self-disclosure of HIV via electronic questionnaire at patient intake is a low-cost option that has not been thoroughly evaluated. We examined 10 years (2009-2019) of patient intake questionnaire data at Moffitt Cancer Center. Self-disclosure of an HIV diagnosis was not uniform, with 36.1% (n = 299, 95% confidence interval [CI] = 32.8% to 39.4%) of 828 patients disclosing. Identification of HIV through this method was highest for anal cancer patients (66.7%, 95% CI = 57.8% to 74.7%). Self-disclosure among patients with hematopoietic malignancies, the most common diagnosis among PLWH at our institution, was lower (19.4%, 95% CI = 14.6% to 25.0%). Patient characteristics associated with HIV self-disclosure included cancer site, natal gender, and race and ethnicity. Findings highlight gaps to motivate future efforts to increase HIV ascertainment prior to initiating cancer care.

Widespread uptake of antiretroviral therapy has effectively improved overall survival in people living with HIV (PLWH) (1,2). This longevity has changed the morbidity profile of PLWH, shifting from opportunistic infections to chronic comorbidities such as cancer (3,4). Accurate information on HIV status during cancer treatment is clinically imperative given higher treatment complication (5-7) and mortality rates following a cancer diagnosis in PLWH (8,9). The Centers for Disease Control and the National Comprehensive Cancer Network Clinical Practice Guidelines in Oncology recommend screening for HIV in health-care settings (10,11). Despite this recommendation, active HIV screening in the oncology setting remains uncommon (12,13). In a study of 18 874 adults initiating care at a large US cancer center between 2004 and 2011, Hwang and colleagues (14) reported a blood-based HIV screening rate of only 18.6%, suggesting that treating oncologists often lack information on this pertinent patient comorbidity when initiating cancer care. Patient self-disclosure of HIV via electronic questionnaire offers an alternative that is minimally invasive, low-cost, and able to be administered at or before first cancer patient interaction, when comorbidity data are most clinically useful. However, HIV self-disclosure has not been thoroughly evaluated in the oncology setting nationally. We therefore examined the prevalence of, and patient features associated with, HIV self-disclosure at a large US cancer center between 2009 and 2019.

We ascertained cancer patient HIV status at Moffitt Cancer Center, a large, National Cancer Institute (NCI)–designated comprehensive cancer center in southwest Florida, between January 2009 and December 2019 using electronic patient questionnaires (EPQs) completed at intake and International Classification of Disease (ICD) codes indicative of HIV (ICD-9: 042-044, 079.53, 795.71, 795.8; ICD-10: V08, B20, B97.35, R75, O98.7, Z21). The EPQ is offered to all patients at intake and, among other topics, includes questions about a prior HIV diagnosis and receipt of therapy for HIV. After completion, EPQ answers are integrated into the electronic health record (EHR) and are accessible by the treating oncologist. In contrast, ICD code assignment does not occur at the time of patient presentation but rather after review of patient charge and billing data and subsequent transfer of assigned comorbidity codes to the Moffitt health research information database. ICD-based HIV ascertainment was estimated to be 75% sensitive and 93% specific at our institution during this time frame (unpublished data). This process occurs on average 6 or more weeks after patient intake. Although the lag time associated with ICD-based HIV ascertainment is not practical to guide oncologists’ treatment plans, the uniformity of retrospective ascertainment makes it an ideal metric against which to compare patterns of EPQ response. Using ICD-based HIV ascertainment as the standard, we computed the proportion (and exact 95% confidence intervals [CIs]) of PLWH and cancer who disclosed their HIV diagnosis on the EPQ. We also compared rates of HIV self-disclosure according to patient characteristics using a 2-sided Pearson χ^2^ test, with a *P* value less than .05 difference considered statistically significant. Scientific and institutional review board approval were obtained from Moffitt Cancer Center to extract and analyze these data. Patient consent to access de-identified data was not required.

In total, 828 PLWH were identified at Moffitt from 2009 to 2019 through either HIV self-disclosure on the EPQ administered at cancer patient intake or assignment of HIV-associated ICD codes after retrospective EHR review. Self-disclosure was not uniform; 36.1% (n = 299, 95% CI = 32.8% to 39.4%) of cancer patients reported their HIV diagnosis on the EPQ. Nineteen patients (2.3%, 95% CI = 1.4% to 3.6%) were uniquely identified because of EPQ self-disclosure, with the remaining 809 having a validated ICD code in the Moffitt health research information system.

Figure 1 displays rates of HIV self-disclosure, ordered by cancer site frequency (height of bars corresponds to cancer count) and grouped by cancers that fall above vs below the average HIV self-disclosure rate of 36.1% (dotted vertical line). The most common cancer observed among PLWH was hematopoietic (heme) malignancies. HIV self-disclosure among heme patients was low (19.4%, 95% CI = 14.6% to 25.0%). Anal cancer was the second most common cancer and was predominantly diagnosed among men. In contrast to heme, 66.7% (95% CI = 57.8-% to 4.7%) of anal cancer patients disclosed their HIV status. Breast cancer was the most common tumor specific to females with HIV at our institution; the rate of HIV self-disclosure among breast cancer patients was close to the overall average (40.0%, 95% CI = 23.8% to 57.8%). Supplementary Table 1 (available online) includes HIV self-disclosure rates by patient features for all cancer types combined. Table 1 highlights patient features that were associated with HIV self-disclosure, both overall and for prevalent cancer sites. Among heme patients, natal gender was associated with HIV self-disclosure; 84.8% of self-disclosers were male, compared with 71.2% of those who did not self-disclose (*P *=* *.04). Self-disclosing heme patients were also marginally more likely to be non-Hispanic White (76.1% among self-report vs 57.6% among no self-report; *P *=* *.10). Among the 129 PLWH and anal cancer, age at diagnosis, natal gender, and race and ethnicity were similar between those who did vs did not self-disclose HIV status. Among breast cancer patients, those who disclosed an HIV diagnosis were far more likely to be African American (85.7%) compared with those who did not (33.3%; *P *=* *.01). Smoking history and highest-attained education level also appeared to be associated with rates of HIV self-disclosure. Unlike the patient characteristics described above, we observed a large amount of missing data for these health behaviors. Nearly all patients who did not disclose an HIV diagnosis also failed to respond to questions on education. However, there was not perfect concordance between answering questions on smoking or education and disclosing HIV status. For example, many patients reported smoking history but did not disclose an HIV diagnosis.

**Figure 1.**
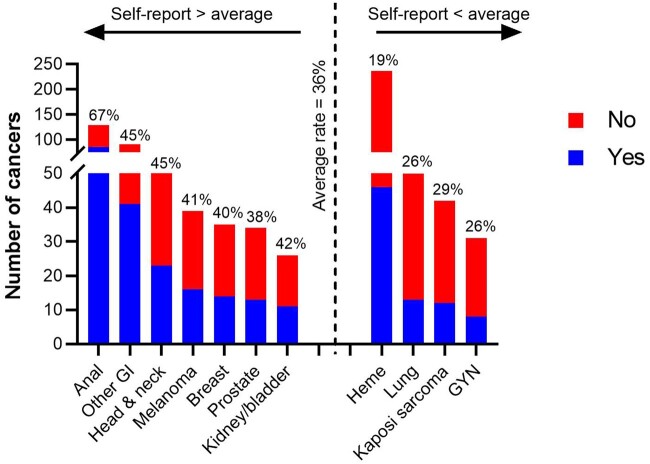
Rates of HIV self-disclosure by cancer site. A total of 828 cancer patients with HIV were identified at Moffitt Cancer Center between 2009 and 2019. Of these, 299 (36.1%) self-reported their HIV status via electronic questionnaire administered at patient intake (**dotted vertical line**). Percentages in the figure correspond to self-report = “yes.” The height of each bar represents the observed cancer count, and cancer sites are grouped according to whether they fall above vs below the average rate of HIV self-disclosure. The figure is limited to cancers with 1) known cancer site and 2) >10 cancers observed. GI = gastrointestinal; GYN = gynecology; Heme = hematopoietic

**Table 1.** Patient features associated with HIV self-disclosure

Cancer type, patient feature	Total, No. (%)	Self-report, No. (%)		*P* ^a^
		Yes	No	
Overall (all cancers)	828	299 (36.1)	529 (63.9)	
Age, y				.20
≤49	380 (45.9)	130 (43.5)	250 (47.3)	
50-69	420 (50.7)	162 (54.2)	258 (48.8)	
≥70	28 (3.4)	7 (2.3)	21 (4.0)	
Race/ethnicity				.17
Non-Hispanic White	526 (63.5)	203 (67.9)	323 (61.0)	
Non-Hispanic Black	173 (20.9)	59 (19.7)	114 (21.6)	
Hispanic	101 (12.2)	30 (10.0)	71 (13.4)	
Other (Asian, mixed race, etc.)	28 (3.4)	7 (2.3)	21 (4.0)	
Natal gender				<.001
Male	609 (73.6)	244 (81.6)	365 (69.0)	
Female	219 (26.4)	55 (18.4)	164 (31.0)	
Education				<.001
High school/GED or less	67 (8.1)	54 (18.1)	13 (2.5)	
Some college or technical/trade school	36 (4.3)	28 (9.3)	8 (1.5)	
College or more	65 (7.9)	46 (15.4)	19 (3.6)	
Missing	660 (79.7)	171 (57.2)	489 (92.4)	
Smoking				<.001
Never	35 (4.2)	1 (0.3)	34 (6.4)	
Former	185 (22. 3)	90 (30.1)	95 (18.0)	
Current	134 (16.2)	65 (21.7)	69 (13.0)	
Missing	474 (57.2)	143 (47.8)	331 (62.6)	
Heme cancers	237 (28.6)	46 (19.4)	191 (80.6)	
Age, y				.25
≤49	129 (54.4)	20 (43.5)	109 (57.1)	
50-69	104 (43.9)	25 (54.3)	79 (41.4)	
≥70	4 (1.7)	1 (2.2)	3 (1.6)	
Race/ethnicity				.10
Non-Hispanic White	145 (61.2)	35 (76.1)	110 (57.6)	
Non-Hispanic Black	39 (16.5)	5 (10.9)	34 (17.8)	
Hispanic	38 (16.0)	3 (6.5)	35 (18.3)	
Other (Asian, mixed race, etc.)	15 (6.3)	3 (6.5)	12 (6.3)	
Natal gender				.04
Male	175 (73.8)	39 (84.8)	136 (71.2)	
Female	62 (26.2)	7 (15.2)	55 (28.8)	
Education				<.001
High school/GED or less	7 (3.0)	5 (10.9)	2 (1.0)	
Some college or technical/trade school	7 (3.0)	4 (8.7)	3 (1.6)	
College or more	17 (7.2)	9 (19.6)	8 (4.2)	
Missing	206 (86.9)	28 (60.9)	178 (93.2)	
Smoking				.05
Never	20 (8.4)	0 (0.0)	20 (10.5)	
Former	50 (21.1)	14 (30.4)	36 (18.8)	
Current	29 (12.2)	4 (8.7)	25 (13.1)	
Missing	138 (58.2)	28 (60.9)	110 (57.6)	
Anal cancer	129 (15.6)	86 (66.7)	43 (33.3)	
Age, y				.99
≤49	70 (54.2)	47 (54.7)	23 (53.5)	
50-69	56 (43.4)	37 (43.0)	19 (44.2)	
≥70	3 (2.3)	2 (2.3)	1 (2.3)	
Race/ethnicity				.52
Non-Hispanic White	93 (72.1)	62 (72.1)	31 (72.1)	
Non-Hispanic Black	21 (16.3)	15 (17.4)	6 (14.0)	
Hispanic	14 (10.9)	9 (10.5)	5 (11.6)	
Other (Asian, mixed race, etc.)	1 (0.0)	0 (0.0)	1 (2.3)	
Natal gender				.54
Male	124 (96.1)	83 (96.5)	41 (95.3)	
Female	5 (3.9)	3 (3.5)	2 (4.7)	
Education				<.001
High school/GED or less	12 (9.3)	12 (14.0)	0 (0.0)	
Some college or technical/trade school	10 (7.8)	10 (11.6)	0 (0.0)	
College or more	12 (9.3)	11(12.8)	1 (0.0)	
Missing	95 (73.6)	53 (61.6)	42 (97.7)	
Smoking				<.001
Never	0 (0.0)	0 (0.0)	0 (0.0)	
Former	29 (22.5)	24 (27.9)	5 (11.6)	
Current	29 (22.5)	25 (29.1)	4 (9.3)	
Missing	71 (55.0)	37 (43.0)	34 (79.1)	
Breast cancer	35 (4.2)	14 (40.0)	21 (60.0)	
Age, y				.50
≤49	15 (42.9)	5 (35.7)	10 (47.6)	
50-69	19 (54.3)	9 (64.3)	10 (47.6)	
≥70	1 (2.9)	0 (0.0)	1 (4.8)	
Race/ethnicity				.01
Non-Hispanic White	15 (42.9)	2 (14.3)	13 (61.9)	
Non-Hispanic Black	19 (54.3)	12 (85.7)	7 (33.3)	
Hispanic	1 (2.9)	0 (0.0)	1 (4.8)	
Other (Asian, mixed race, etc.)	0 (0.0)	0 (0.0)	0 (1.0)	
Natal gender				.51
Male	2 (5.7)	0 (0.0)	2 (9.5)	
Female	33 (94.3)	14 (100.0)	19 (90.5)	
Education				.16
High school/GED or less	5 (14.3)	4 (28.6)	1 (4.8)	
Some college or technical/trade school	2 (5.7)	1 (7.1)	1 (4.8)	
College or more	6 (17.1)	3 (21.4)	3 (14.2)	
Missing	22 (62.9)	6 (42.9)	16 (76.2)	
Smoking				.82
Never	0 (0.0)	0 (0.0)	0 (1.0)	
Former	7 (20.0)	3 (21.4)	4 (19.0)	
Current	6 (17.1)	3 (21.4)	3 (14.2)	
Missing	22 (62.9)	8 (57.1)	14 (66.7)	

a We compared rates of HIV self-disclosure according to patient characteristics using a 2-sided Pearson χ^2^ test, with *P *<* *.05 differences considered statistically significant. Heme = hematopoietic; GED = tests of General Education Development.

Cancer is increasingly common in the aging HIV population, but there has been little success in uniformly identifying PLWH through blood-based screening at oncology centers. This is problematic given the adverse impact of HIV on cancer patient outcomes (8,9), making it crucial information for oncologists’ treatment plans. Self-report via electronic questionnaire offers a low-cost method to improve ascertainment of HIV status. Our data from 828 patients at a large, NCI-designated comprehensive cancer center indicate that rates of self-disclosure of an HIV diagnosis at cancer patient intake (36.1%), although not uniform, exceed blood-based HIV screening rates in the largest oncology center report to date (18.6%) (14). We encourage HIV ascertainment, whether self-disclosure or blood-based screening, but posit that self-disclosure may be a viable option for cancer centers that are not part of health systems that provide infectious disease care but do utilize intake questionnaires (remote or in-person) that could be modified at little cost to be HIV inclusive. The patient features we identified as being associated with HIV self-disclosure could guide future efforts to improve ascertainment of HIV status at the time of cancer treatment initiation. For example, low rates of self-report in select clinical programs and/or patient groups could help target efforts to improve acceptability and access to HIV-inclusive EPQ administration or pilot test EHR-linked provider alerts to discuss cancer patient HIV status. Such efforts could help achieve a more accurate description of the HIV burden in oncology centers nationally.

## Funding

This work was funded through the Biostatistics Core Facility at Moffitt Cancer Center under the Cancer Center Support Grant (NCI P30 CA076292, PI: Cleveland).

## Notes

**Role of the funder:** The funder had no role in study design, data collection and analysis, decision to publish, or preparation of the manuscript.

**Disclosures:** The authors have no conflicts of interest relevant to this publication to disclose.

**Author contributions:** Fuzzell: writing—original draft, writing—review & editing, visualization, project administration, formal analysis; Vadaparampil: conceptualization, writing—review & editing; Giuliano: writing—review & editing; Liu: data curation, formal analysis, writing—review & editing; Coghill: writing—review & editing, visualization, formal analysis, methodology, conceptualization, supervision.

**Prior presentations:** Similar findings related to this manuscript were presented virtually via a poster at the 2020 annual meeting of the American Society of Preventive Oncology.

## Data Availability

The data underlying this article cannot be shared publicly due to the privacy of patients at the institution. The corresponding author will consider reasonable requests for de-identified data.

## Supplementary Material

pkab058_Supplementary_Data
