# Supplementary material for: Patterns of HIV Self-Disclosure in the Oncology Setting
Source: JNCI Cancer Spectr. 2021 Jun 4;5(4):pkab058. doi: 10.1093/jncics/pkab058 (PMC8328009; doi:10.1093/jncics/pkab058)
Supplement: pkab058_Supplementary_Data [file pkab058_supplementary_data.pdf]

Supplementary Table 1. Rates of HIV self-disclosure by cancer patient features**.**

Patient features	Total No.	Self-report yes, No. (%)
All cancers	828	299 (36.1)
Age, y		
≤49	380	130 (34.2)
50-69	420	162 (38.6)
≥70	28	7 (25.0)
Race/Ethnicity		
Non-Hispanic White	526	203 (38.6)
Non-Hispanic Black	173	59 (34.1)
Hispanic	101	30 (29.7)
Other (Asian, Mixed Race, etc.)	28	7 (25.0)
Natal Gender		
Male	609	244 (40.1)
Female	219	55 (25.1)
Education		
< High School or high school GED	67	54 (80.6)
Some college or tech/trade school	36	28 (77.8)
College or more	65	46 (70.8)
Missing	660	171 (25.9)
Smoking		
Never	35	1 (2.9)
Former	185	90 (48.6)
Current	134	65 (48.5)
Missing	474	143 (30.2)
